# Supplementary material for: Biosorption Characteristic and Cytoprotective Effect of Pb2+, Cu2+ and Cd2+ by a Novel Polysaccharide from Zingiber strioatum
Source: Molecules. 2022 Nov 19;27(22):8036. doi: 10.3390/molecules27228036 (PMC9696034; doi:10.3390/molecules27228036)
Supplement: Supplementary file 1 [file molecules-27-08036-s001.zip › molecules-2023013-supplementary.pdf]

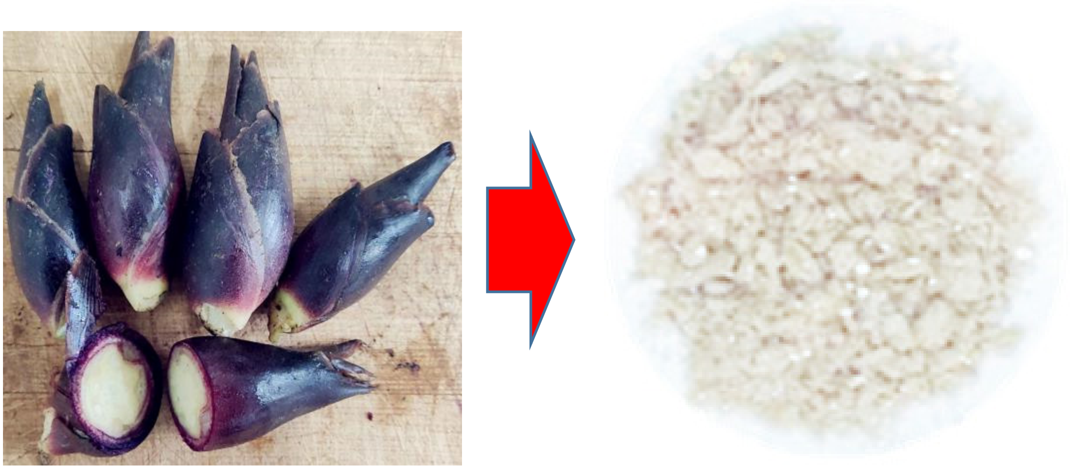

Figure S1. Fresh *Zingiber strioatum* and polysaccharide from *Zingiber strioatum*(ZSP).

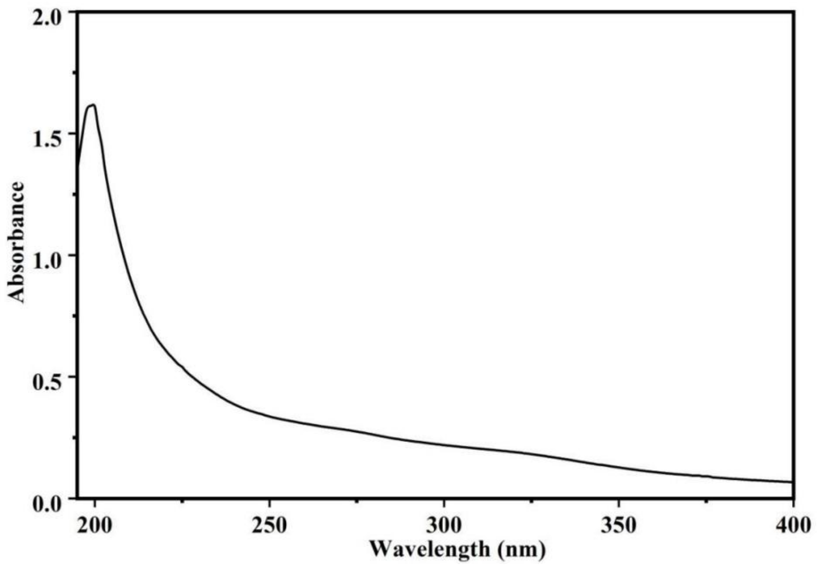

Figure S2. The ultraviolet scan spectrum of ZSP.

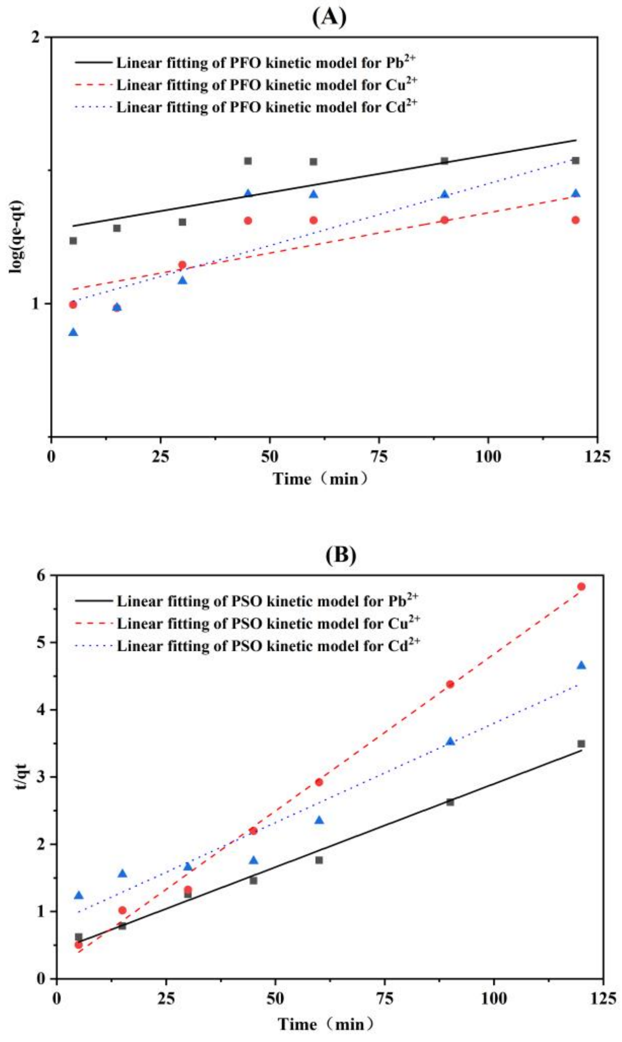

Figure S3. (A) pseudo-first order kinetic model and (B) pseudo-second order kinetic model for the Pb^{2+}, Cu^{2+}, Cd^{2+} adsorption on the ZSP.

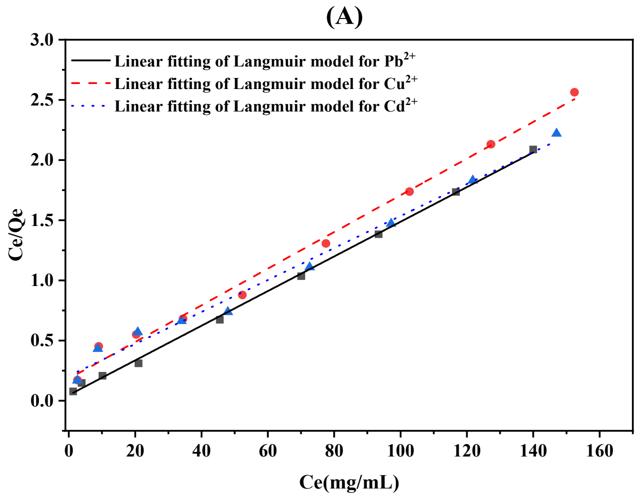

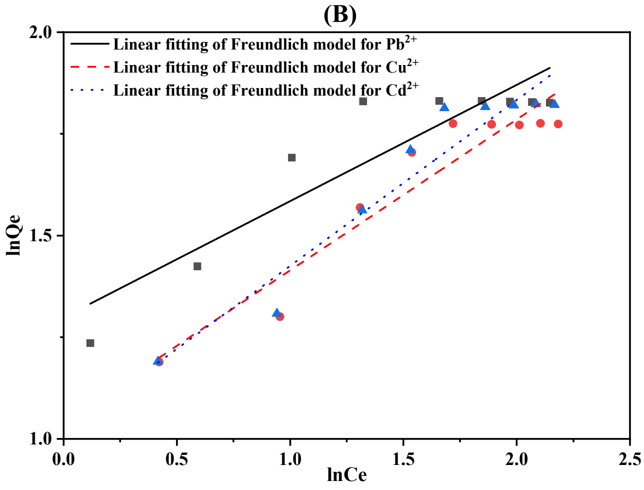

Figure S4. (A) Langmuir isotherm model and (B) Freundlich isotherm model for the Pb^{2+}, Cu^{2+}, Cd^{2+} adsorption on the ZSP
